# Seroepidemiological survey of *Neospora caninum* and its risk factors in farm dogs in Nakuru district, Kenya

**DOI:** 10.14202/vetworld.2016.1162-1166

**Published:** 2016-10-30

**Authors:** Tequiero Abuom Okumu, John Njenga Munene, James Wabacha, Victor Tsuma, John Van Leeuwen

**Affiliations:** 1 Department of Clinical Studies, Faculty of Veterinary Medicine, University of Nairobi, Nairobi City, Kenya; 2 Department of Clinical Studies, Faculty of Veterinary Medicine and Surgery, Egerton University, Njoro, Nakuru County, Kenya; 3 AQ1 African Union Inter Africa Bureau of Animal Resources, Nairobi, Kenya; 4 Department of Health Management, Atlantic Veterinary College, University of Prince Edward Island, Canada

**Keywords:** cross-sectional study, dogs, Kenyan dairy farms, Neosporosis

## Abstract

**Aim:**

The objective of this study was to determine the seroprevalence of *Neospora caninum* (NC) and its risk factors in farm dogs in Kenya.

**Materials and Methods:**

As part of a longitudinal study on dairy cattle abortion in 2010 in Kenya, serum samples were collected from 84 dogs in 53 randomly selected dairy cattle farms to determine the seroprevalence and risk factors of seropositivity for NC.

**Results:**

15 (17.9%) of the dogs were seropositive to NC antibodies, and at least one seropositive dog was found in 12 (22.6%) of the 53 farms. The final multivariable logistic regression model identified free-roaming as the only factor significantly associated with seropositivity (odds ratio=4.48; p=0.03).

**Conclusion:**

The findings of this study indicate that canine neosporosis does exist in Kenya and that farmers should restrict their dogs from roaming to reduce the risk of their dogs becoming a reservoir for NC. More studies need to be carried out to determine the reproductive effects of NC on dairy cattle in Kenya.

## Introduction

Neosporosis in dogs is caused by *Neospora caninum* (NC), a coccidian parasite that was first described in 1984 [1]. The parasite is related to *Toxoplasma gondii* [2]. While canids act as definitive and intermediate hosts of this parasite [1,3-5], many other mammalian species have been described as intermediate hosts of NC. Among these mammals are cattle, sheep, goats, deer, moose, water buffalo, camels, and wild birds [3,6]. The rate of vertical transmission in canine neosporosis has been reported to be low; therefore, horizontal transmission through direct ingestion of tachyzoites in placentae, aborted fetuses, or improperly cooked meat with tissue cysts is thought to be the main route of spread within canids [1,7].

The impacts of canine neosporosis are two-fold: In the dog population, congenital infection can lead to neuromuscular defects and mortality [8]; and NC oocysts shed in canid feces can also lead to horizontal transmission to ruminants, especially cattle, leading to reproductive losses, such as abortion since the parasite is fetopathogenic [1,4,9-12]. A recent systematic review reports the median estimate of the global economic impact of NC infections/abortions to be $1.3 billion per annum [13].

There are reports on the occurrence of canine neosporosis in various parts of the world, with seroprevalence levels ranging from 0% to 32% being reported [1,2,8,14-17]. A previous Kenyan study reported that out of 140 dogs screened, none was seropositive to NC [14]. However, this Kenyan population of dogs was a feral dog population, and therefore, not necessarily representative of the farm dog population. Further research is needed on NC in dogs in Kenya, especially among the farm dog population.

There is limited knowledge on the risk factors associated with dogs with NC infections. An Italian study reported that the prevalence of neosporosis in rural and urban dogs was 26% and 14.6%, respectively. The same study also reported that the prevalence in free-roaming dogs (35.8%) was double compared to confined dogs (17.3%) and that the 13.9% prevalence in young dogs <1.5-year-old was less than in older dogs more than 5-year-old, at 37.8% prevalence [8]. A Brazilian study [7] reported similar findings, and in addition, reported higher prevalences in older dogs, dogs fed uncooked meat, and mixed-breed dogs. However, further research is needed to verify which of these risk factors are valid in the African and/or Kenyan context and to quantify the amount of risk the factors represent in this context.

The objective of this study was to determine the seroprevalence of NC and risk factors associated with NC infection in farm dogs in Nakuru District, Kenya. This research was part of a longitudinal study on infectious causes of abortion in dairy cattle.

## Materials and Methods

### Ethical approval

This study was approved by the Faculty of Veterinary Medicine Postgraduate Committee of the University of Nairobi.

### Study population and sampling

The study was carried out in 2010 in the former greater Nakuru District of Kenya, a dairy cattle-rearing area. A list of dairy cattle farms was collected from the Nakuru District Animal Production Office. Using a random number table, 61 farms were randomly selected and agreed to participate in the study, with 6 other farms refusing to participate. Among these farms, the study population for this portion of the research project included farmers meeting three inclusion criteria: (1) Consent to participate in the study; (2) had at least one dog resident on the farm; and (3) ability to provide their dog for a blood sample during the farm visit.

During farm visits, a questionnaire was used to collect data on each dog’s age (categorized as <3 years old or >3 years), breed (purebred or crossbred), sex (male or female), diet (allowed to eat abattoir and aborted bovine fetuses and placentae or not), rurality (peri-urban farms around Naivasha [an urban area in Nakuru District] or not), and ability to roam free (confined or free-roaming).

### Sample collection and laboratory analysis

Farm dogs were physically restrained in a humane manner and serum samples collected from the cephalic veins. After blood collection into plain Vacutainer tubes, the blood was allowed to clot and then put in a cooler with an ice pack (4°C) until it was transported back to the laboratory for processing within 4 h. After centrifuging the blood, serum was harvested into labeled Eppendorf tubes and stored at −20°C awaiting screening for antibodies to NC after all serum samples were collected.

At the Faculty of Veterinary Medicine at the University of Nairobi, the serum samples were assayed in a commercial ELISA antibody test kit (Herdcheck^®^ NC antibody test kit, IDEXX Laboratories, Switzerland AG) to test the serum for exposure to NC, with some recommended modifications [18].

### Statistical analysis

Data from the survey and serological status were entered and stored in Excel v2007 (Microsoft Corporation, Redmond WA, USA). The data were screened for any entry errors. The data were imported into Genstat^®^ 13^th^ edition, service pack two, for analysis (VSN International, Hemel Hempstead, UK).

Descriptive statistics, including prevalence, correlations, and 95% confidence intervals (CI), were determined for the outcome and risk factor variables of interest from the survey. In Genstat^®^, the Pearson’s Chi-square test was used to determine unconditional associations between predictor variables and NC seropositivity, with the significance set at p<0.1.

Multiple variable logistic regression was carried out to model the effects of potential risk factors on the seropositivity of NC in farm dogs in Nakuru District while controlling for the effects of confounding of other variables in the model. A backward elimination procedure was used to build the model of main effects on the seropositivity outcome. Factors that were found significant (p≤0.05) were retained in the final model. Odds ratios (ORs) and 95% CI, as a measure of the strength of association between the significant variables (p<0.05) and the outcome, were calculated.

## Results

Of the 61 farms selected to participate in the study, samples were collected from dogs on 53 farms. Dogs from 8 farms could not be traced during the scheduled visits due to roaming. A total of 84 dogs were sampled on the farms. The number of dogs on each of the farms ranged from 1 to 3. The farms had predominantly Friesians (68% of farms) that were on pasture (63% of farms), varying in size from 1 to 170 milking cows.

Overall, 15 of the 84 (17.9%; 95% CI: 9.7-26.1) dogs sampled were positive for antibodies to NC. At least one seropositive dog was found in 12 of 53 farms (22.6%; 95% CI: 11.3-33.9), though no cases of clinical neosporosis were encountered in the dog population.

The seroprevalence to NC in relation to other variables is shown in Table-1. On univariable analysis, the seroprevalence of NC in free-roaming dogs (36.4%; 95% CI: 16.3-56.5) was significantly higher than in confined dogs (11.3%; 95% CI: 3.4-19.2). There were no significant differences in seroprevalence (p>0.05) for the remaining variables, although marginal significance was achieved for dogs being fed fetuses, and rurality (0.1>p>0.05).

**Table-1:** Descriptive statistics and univariable associations between seropositivity for NC and epidemiological data for 84 dogs on 53 dairy farms in Nakuru District, Kenya in 2010.

Epidemiological data	Examined animals	# Positive (%) to *Neospora*	OR	p value
Free-roaming				
No	62	7 (11.3)		
Yes	22	8 (36.4)	4.48	0.03
Fetal-feeding				
No	12	4 (33.3)		
Yes	61	10 (16.4)		
Unknown	11	1 (9.1)	0.39	0.07
Sex				
Female	19	2 (10.5)		
Male	65	13 (20.0)	2.12	0.11
Breed				
Cross-breed	79	14 (17.7)		
Pure-breed	5	1 (20.0)	1.16	0.30
Age				
<3 years	54	9 (16.7)		
≥3 years	30	6 (20.0)	0.80	0.20
Rurality				
Peri-urban dogs (Naivasha)	11	1 (9.1)		
Rural dogs (Rongai, Molo and Njoro areas)	73	14 (19.2)	2.84	0.08

NC=*Neospora caninum*, OR=Odds ratio

In the final model, free-roaming was significantly positively associated with seropositivity for NC in dogs on dairy cattle farms in Nakuru District (OR=4.48; p=0.03; 95% CI: 1.39-14.49). No other predictor variables remained significant in the final model.

## Discussion

This is the first positive report of NC in farm dogs in Kenya, with a seroprevalence of 17.9%. A previous study [14] had found no seropositive dogs in Kenya. This was probably due to differences in the dog populations under study; the dogs in the previous study [14] were feral dogs, and therefore, were not specifically associated with farms, whereas the dogs in the current study were all farm dogs. This domestic/agricultural source of dogs increased their chances of interacting with cattle who are the main intermediate hosts of this canine parasite [19]. The seroprevalence in the current study was within the range reported in other studies (0-31%) [1,8,16].

Congenital infection through transplacental infection, and horizontal transmission through direct ingestion of oocysts in placentae, tissue cysts, or improperly cooked meat are considered the main routes of spread of NC in dogs [7]. Free-roaming was the only factor significantly associated with seropositivity to NC. Indeed, the NC seroprevalence in free-roaming farm dogs was more than three times higher than in confined farm dogs, likely due to increased chances of exposure to potential sources of infection such as aborted fetuses and placenta from cattle, as well as other intermediate hosts. This finding was in agreement with a Brazilian study which found an OR of 2.2 for lack of confinement and canine neosporosis [7]. Other studies have also reported higher NC seroprevalences in free-roaming dogs relative to other groups [20,21] while controlling for whether the dogs came from a rural, urban, or peri-urban setting. Our study was designed to control for setting in two ways: (1) in only sampling farm dogs, although some of the dogs did come from peri-urban farms around Naivasha and (2) the use of multiple variable logistic regression which is designed to examine and control for other variables, such as setting.

In our study, the prevalence of NC was slightly higher in the rural areas of the study (Molo, Njoro and Rongai) at 19.2%, as opposed to Naivasha at 9.1%, which is a more urban setting, although this difference was not statistically significant (p>0.05). Similar differences between rural and urban dog populations have been reported [7,16,20,22], and this may be due to their greater likelihood to encounter cattle offal.

In our study, there was a trend for seroprevalence of NC to increase with age, as reported in other studies [7,8,15,21,22], although this difference was not statistically significant (p>0.05). The increased prevalence in older dogs may be due to their cumulative risk of exposure throughout their life relative to younger dogs thus suggesting horizontal transmission plays a major role in the epidemiology of canine neosporosis. It has been reported that some infected young animals may fail to seroconvert or may have a slow rate of seroconversion when infected by NC [3]. This delayed seroconversion may also have led to this trend of lower prevalence rates in younger dogs.

Despite many dogs being fed fetal parts from the abattoirs, their seroprevalence was marginally lower than those not fed fetal parts, though the association was not statistically significant. This difference may be attributed to the fact that these fetal parts may have been cooked before being fed to the dogs (as mentioned by some farmers), thus reducing their infectivity. Cooking of fetal parts was not asked among the initial farmers, making it impossible to check for interactions between cooking and feeding fetal parts. The owners mentioned that the dogs not fed fetal parts were more likely to scavenge on carcasses, as well as hunt birds and rodents, which have also been reported as potential sources of infection [23]. These dogs not fed fetal parts were also more likely to roam in search for food, thus further increasing their risk of exposure, as discussed earlier.

A higher NC seroprevalence in male dogs relative to bitches has been reported [7,16,21]. In our study, the prevalence of antibodies to NC was somewhat higher in males than females, though the association was not statistically significant. This difference may be due to the fact that more people were likely to confine females to avoid unwanted breeding, thus reducing their chances of getting infected with NC.

Most studies on canine neosporosis have been based on the serological status of sampled dogs due to the low and intermittent shedding of oocysts by the host, making oocysts difficult to detect in field conditions [2,14,23]. The main limitations to the use of serological tests in the diagnosis of canine neosporosis are that it takes 2-3 weeks for antibodies to be detectable in recently infected animals and the fact that some dogs shedding fecal oocysts actually do not have detectable antibody titers [24]. As a result, some false positives and negatives may occur. Furthermore, risk factors of infection prevalence are usually less informative for understanding factors of transmission than infection incidence studies because prevalence is a function of both incidence and duration, making risk factor studies based on prevalence harder to interpret [25]. Therefore, a risk factor incidence study of seroconversion of dogs to NC or a study of oocyst-shedding dogs (recently infected) would be a useful addition to the literature.

## Conclusion

Our study suggests that canine neosporosis is common in Kenyan dairy cattle farms and that lack of confinement appears to predispose dogs to infection. Measures should be taken to restrict free-roaming behavior in dogs which should reduce exposure of dogs to potential sources of infection, such as through placentae and fetuses. With confirmed diagnosis of NC in Kenyan farm dogs, fecal contamination of water and feed sources for cattle, by potential definitive hosts such as farm dogs, should be avoided to prevent horizontal NC transmission. In addition, studies should be carried out to determine the reproductive effects of NC on cattle in Kenya.

## Authors’ Contributions

TAO conducted the field and laboratory work. In addition, he participated in the study design, did the data analysis and preparation of this manuscript. JNM, JWJ, VT, and JVL were involved in the conception of the study, study design and the writing of this manuscript.
